# OSAT: a tool for sample-to-batch allocations in genomics experiments

**DOI:** 10.1186/1471-2164-13-689

**Published:** 2012-12-10

**Authors:** Li Yan, Changxing Ma, Dan Wang, Qiang Hu, Maochun Qin, Jeffrey M Conroy, Lara E Sucheston, Christine B Ambrosone, Candace S Johnson, Jianmin Wang, Song Liu

**Affiliations:** 1 Department of Biostatistics and Bioinformatics, Roswell Park Cancer Institute, Buffalo, NY, 14263, USA; 2 Department of Biostatistics, SUNY University at Buffalo, Buffalo, NY, 14214, USA; 3 Cancer Genetics, Roswell Park Cancer Institute, Buffalo, NY, 14263, USA; 4 Cancer Prevention and Control, Roswell Park Cancer Institute, Buffalo, NY, 14263, USA; 5 Pharmacology and Therapeutics, Roswell Park Cancer Institute, Buffalo, NY, 14263, USA

## Abstract

**Background:**

Batch effect is one type of variability that is not of primary interest but ubiquitous in sizable genomic experiments. To minimize the impact of batch effects, an ideal experiment design should ensure the even distribution of biological groups and confounding factors across batches. However, due to the practical complications, the availability of the final collection of samples in genomics study might be unbalanced and incomplete, which, without appropriate attention in sample-to-batch allocation, could lead to drastic batch effects. Therefore, it is necessary to develop effective and handy tool to assign collected samples across batches in an appropriate way in order to minimize the impact of batch effects.

**Results:**

We describe OSAT (Optimal Sample Assignment Tool), a bioconductor package designed for automated sample-to-batch allocations in genomics experiments.

**Conclusions:**

OSAT is developed to facilitate the allocation of collected samples to different batches in genomics study. Through optimizing the even distribution of samples in groups of biological interest into different batches, it can reduce the confounding or correlation between batches and the biological variables of interest. It can also optimize the homogeneous distribution of confounding factors across batches. It can handle challenging instances where incomplete and unbalanced sample collections are involved as well as ideally balanced designs.

## Background

A sizable genomics study such as microarray often involves the use of multiple batches (groups) of experiment due to practical complication. The systematic, non-biological differences between batches in genomics experiment are referred as batch effects. Batch effects are wide-spread occurrences in genomic studies, and it has been shown that noticeable variation between different batch runs can be a real concern, sometimes even larger than the biological differences [1-5]. Without sound experiment designs and statistical analysis methods to handle batch effects, misleading or even erroneous conclusions could be made. This especially important issue is unfortunately often overlooked, partially due to the complexity and multiple steps involved in genomics studies.

To minimize the impact of batch effects, a careful experiment design should ensure the even distribution of biological groups and confounding factors across batches. It would be problematic if one batch run contains most samples of a particular biological group. In an ideal genomics design, the groups of the main interest, as well as important confounding variables should be balanced and replicated across the batches to form a Randomized Complete Block Design (RCBD) [6-8]. It makes the separation of the real biological effect of our interests and effects by other confounding factors statistically more powerful.

However, despite all best effort, it is often than not that the collected samples are not complying with the original ideal RCBD design. This is due to the fact that these studies are mostly observational or quasi-experimental since we usually do not have full control over sample availability [1]. In clinical genomics study, samples may be rare, difficult or expensive to collect, irreplaceable or fail QC before profiling. The resulted unbalance and incompleteness nature of sample availability in genomics study, without appropriate attention in sample-to-batch allocation, could lead to drastic batch effects. Therefore, it is necessary to develop effective and handy tool to assign collected samples across batches in an appropriate way in order to minimize the impact of batch effects.

We developed OSAT to facilitate the allocation of collected samples into different batches in genomics studies. OSAT is not aimed to be a software for experimental design carried out before sample collection, rather, it is developed to fulfill the needs arise from some practical limitations occurring in the genomics experiments. Specifically, OSTA is developed to address one practical issue in genomics studies – when the available experimental samples ready to be profiled in the genomics instruments are collected, how should one allocate these samples to different batches in a proper way to achieve an optimal setup minimizing the impact of batch effects at the genomic profiling stage? With a block randomization step followed by an optimization step, it produces setup that optimizes the even distribution of samples in groups of biological interest into different batches, reducing the confounding or correlation between batches and the biological variables of interest. It can also optimize the even distribution of confounding factors across batches. OSAT can handle challenging instances where incomplete and unbalanced sample collections are involved as well as ideal balanced RCBD.

## Results

### Datasets

An exemplary data is used for demonstration. It represents samples from a study where the primary interest is to investigate the expression differentiation in case versus control groups (variable SampleType). Two additional variables, Race and AgeGrp, are clinically important variables that may have impact on final outcome. We consider them as confounding variables. A total of 576 samples are included in the study, with one sample per row in the example file. As shown in Additional file 1: Table S1–S2, none of the three variables are characterized by balanced distribution.

### Comparison of different sample assignment algorithms

The default algorithm implemented in OSAT will first block three variables considered (*i.e*., SampleType, Race and AgeGrp) to generate a single initial assignment setup, and then identify the optimal one with most homogeneous cross-batch strata distribution through shuffling the initial setup. Alternatively, if blocking the primary variable (*i.e.*, SampleType) is the most important and the optimization of the other two variables is less important (but desired), a different algorithm implemented in OSATcan be used. It works by first blocking SampleType only to generate a pool of assignment setups, and then select the optimal one with most homogeneous cross-batch strata (*i.e.*, SampleType, Race and AgeGrp) distribution.

**Figure 1:**
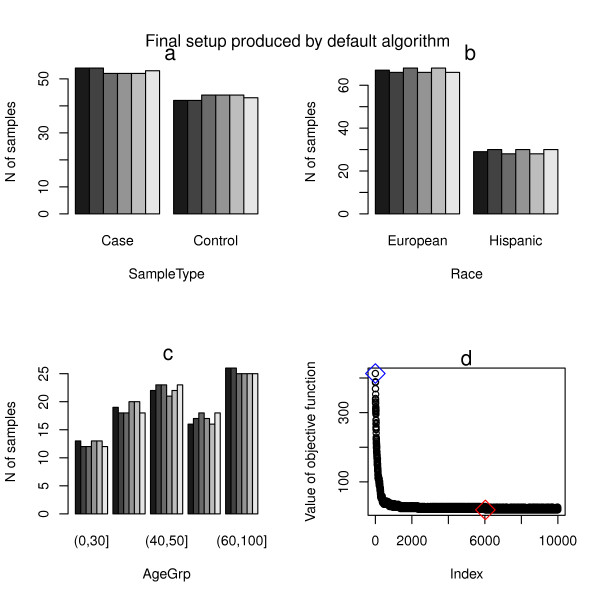
**Summary of final setup produced by the default algorithm. a)** the distribution of SampleType characteristic across the plates; **b)** the distribution of Race characteristic across the plates; **c)** the distribution of AgeGrp characteristic across the plates; **d)** the index of optimization steps versus value of the objective function. The blue diamond indicates the starting point, and the red diamond marks the final optimized setup.

**Figure 2:**
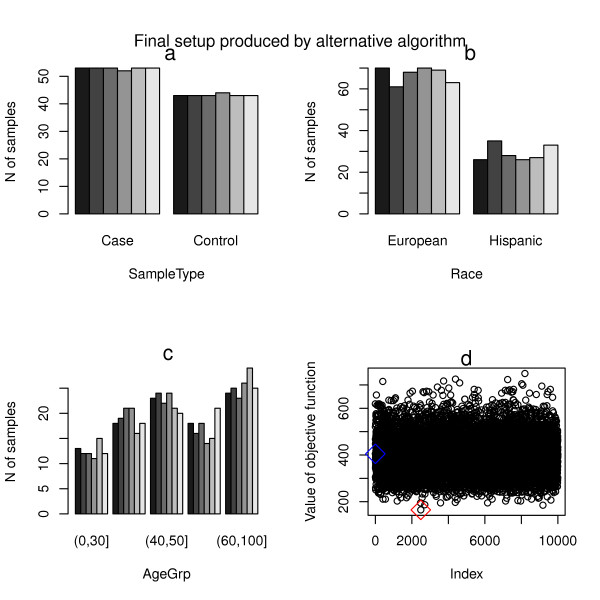
**Summary of final setup produced by the alternative algorithm. a)** the distribution of SampleType characteristic across the plates; **b)** the distribution of Race characteristic across the plates; **c)** the distribution of AgeGrp characteristic across the plates; **d)** the index of generated setups versus value of the objective function. The blue diamond indicates the first setup generated, and the red diamond marks the final selected setup.

As shown in Figure 1a-c, the final setup produced by the default algorithm is characterized by relatively uniform distribution of all three variables across the batches. Pearson’s χ^2^ test examining the association between batches and each of the variables considered indicate that all there variables considered are highly uncorrelated with batches (p-value > 0.99, Table 1). On the other hand, as shown in Figure 2a-c, the final setup produced by the alternative algorithm is characterized by almost perfectly uniform distribution of SampleType variable (with small variation only due to the inherent limitation of the starting data such as unbalanced sample collection), with the uniformity of the other two variables not included in block randomization step decreased. Pearson's χ^2^ test (Table 1) shows that the resulting chi-square for SampleType decreases while those for Race and AgeGrp increase, indicating the tradeoff in prioritizing variable of primary interest for block randomization. Nevertheless, as shown in Figure 1d and Figure 2d, both algorithms produce final setups which show more homogeneous cross-batch strata distribution than the corresponding starting ones.

**Table 1:** Comparison of sample assignment by two algorithms implemented in OSAT and an undesired sample assignment through complete randomization

		**Default algorithm**		**Alternative algorithm**		**An undesired setup through complete randomization**	
		**(optimal.shuffle)**		**(optimal.block)**			
**Variable**	***DF***	***Chi-square***	***P value***	***Chi-square***	***P value***	***Chi-square***	***P value***
**SampleType**	5	0.2034518	0.9990763	0.03507789	0.9999879	13.25243	0.021124664
**Race**	5	0.2380335	0.9986490	3.68541503	0.5955359	14.22455	0.014244218
**Age_grp**	20	0.8138166	1.0000000	5.08147313	0.9996856	39.75020	0.005371387

**Figure 3:**
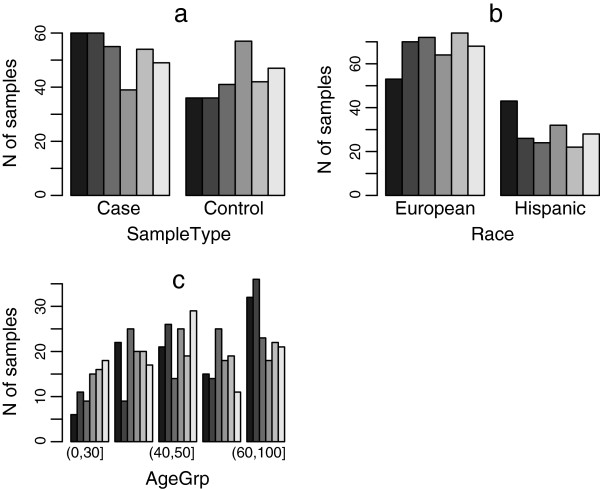
**Summary of an undesired setup produced by complete randomization. a)** the distribution of SampleType characteristic across the plates; **b)** the distribution of Race characteristic across the plates; **c)** the distribution of AgeGrp characteristic across the plates.

Simply performing complete randomizations might lead to undesired sample-to-batch assignment, especially for unbalanced and/or incomplete sample sets. In fact, there is substantial chance that variables will be statistically dependent on batches if a complete randomization is carried out, especially for incomplete and/or unbalanced sample collections. As shown in Figure 3, an undesired setup can be produced through complete randomization of sample-to-batch assignment. The Pearson's χ^2^ tests indicate all three variables are statistically dependent on batches with p-values < 0.05 (Table 1).

## Conclusions

Genomics experiments are often driven by the availability of the final collection of samples which might be unbalanced and incomplete. The unbalance and incompleteness nature of sample availability thus calls for the development of effective tools to assign collected samples across batches in an appropriate way in order to minimize the impact of batch effects at the genomics experiment stage. OSAT is developed to facilitate the allocation of collected samples to different batches in genomics study. With a block randomization step followed by an optimization step, it produces setup that optimizes the even distribution of samples in groups of biological interest into different batches, reducing the confounding or correlation between batches and the biological variables of interest. It can also optimize the homogeneous distribution of confounding factors across batches. While motivated to handle challenging instances where incomplete and unbalanced sample collections are involved, OSAT can also handle ideal balanced RCBD.

Partly due to its simplicity in implementation, complete randomization has been frequently used in the sample assignment step of experiment practice. When sample size is large enough, randomized design will be close to a balanced design. However, simple randomization could lead to undesirable imbalanced design where efficiency and confounding might be an issue after the data collection. As we demonstrated in the manuscript, simply performing randomizations might lead to undesired sample-to-batch setup showing batch dependence, especially for unbalanced and/or incomplete sample sets which doesn’t comply with the original ideal design. OSAT package is designed to avoid such scenario, by providing a simple pipeline to create sample assignment that minimizes the association between sample characteristics and batches. The software was implemented in a flexible way so that it can be adopted by genomics practitioner who might not be specialized in experiment design.

It should be emphasized that although the impact of batch effect on genomics study might be minimized through proper design and sample allocation, it may not be completely eliminated. Even with perfect design and best effort in all stages of experiment including sample-to-batch assignment, it is impossible to define or control all potential batch effects. Many statistical methods have been developed to estimate and reduce the impact of batch effect at the data analysis stage (*i.e.,* after the experiment part is done) [1,9-12]. It would be helpful that analytic methods handling batch effects are employed in all stages of a genomics study, from experiment design to data analysis.

Experimental design has been applied in many areas, with methods being tailored to the needs of various fields. A collection of R packages for experimental design is available at http://cran.r-project.org/web/views/ExperimentalDesign.html. Many of these existing experiment design software work for ideal situation (i.e., before sample collection) where the sample size is fixed and/or model is specified. For example, the software in above link includes optimal design (e.g. *AlgDesign*, requiring model specification), orthogonal arrays for main effects experiments (e.g., function *oa.design*, constrained by sample size/number of factors), factorial 2-level designs (e.g., Package *FrF2*, particularly important in industrial experimentation), and etc. We developed OSAT to facilitate the allocation of collected samples into different batches in genomics studies. Our software implements the general experiment design methodology to achieve the optimal sample-to-batch assignment in order to minimize the impact of batch effects. It is specifically used in the profiling stage of a genomics study when the available experimental samples ready to be profiled in the genomics instruments are collected. It provides predefined batch layout for some of the most commonly used genomics platforms. Written in a modularized style in the open source R environment, it provides the flexibility for users to define the batch layout of their own experiment platform, as well as optimization objective function for their specific needs, in sample-to-batch assignment in order to minimize the impact of batch effects. To our best knowledge, there is no other tool for this important utility within the framework of Bioconductor.

## Methods

### Methodology

The current version of OSAT provides two algorithms for creation of sample assignment across the batches based on the principle of block randomization, which is an effective approach in controlling variability from nuisance variables such as batches and its interaction with variables of our primary interest [6-8,13]. Both algorithms are composed of a block randomization step and an optimization step. The default algorithm (implemented in function *optimal.shuffle*) sought to first block all variables considered to generate a single initial assignment setup, then identify the optimal one which minimizes the objective functions (*i.e.,* the one with most homogeneous cross-batch strata distribution) through shuffling the initial setup. The alternative algorithm (implemented in function *optimal.blcok*) sought to first block specified variables (*e.g.*, list of variables of primary interests) to generate a pool of assignment setups, then select the optimal one which minimize the objective functions based on all variables considered (including those variables which are not included in the block randomization step). A detailed description is provided as below.

By combining the variables of interest, we can create a unified variable with its levels based on all possible combinations of the levels of the variables involved. Assuming there are a total of *s* levels in the unified variable (referred as optimization strata in this package) with *S*_*j*_ samples in each stratum, *j* = 1 … *s*, and assuming we have *m* batches with *B*_*i*_, *i = 1… m* wells available in each batch. In an ideal balanced RCBD experiment, we have equal sample size in each strata: *S*_*1*_*= …= S*_*s*_*= S,* and each batch includes the same number of available wells, *B*_*1*_*= … = B*_*m*_*= B*, with equal number of samples from each sample strata.

The expected number of sample from each stratum to each batch is denoted as *E*_*ij*_. One can split it to its integer part and fractal part as

$$E_{\mathrm{ij}}=\frac{B_{i}j}{\sum_{i}B_{i}}=\left\lfloor E_{\mathrm{ij}}\right\rfloor +\delta_{\mathrm{ij}}$$

where ⌊*E*_*ij*_⌋ is the integer part of the expected number and *δ*_*ij*_ is the fractal part. In the case of equalbatch size, it reduces to $\left\lfloor E_{\mathrm{ij}}\right\rfloor =\frac{S_{j}}{m}$ . When we have RCBD, all *δ*_*ij*_ are zero.

For an actual sample assignment

$$\begin{array}{c} B_{1}\\ \vdots \\ B_{m} \end{array}\begin{array}{c} \begin{array}{ccc} S_{1} & \ldots & S_{s} \end{array}\\ \left(\begin{array}{lll} n_{11} & \ldots & n_{1s}\\ \vdots & \ddots & \vdots \\ n_{m1} & \ldots & n_{\mathrm{ms}} \end{array}\right) \end{array}$$

where *n*_*ij*_ is the number of sample in each optimization strata from an actual sample assignment. Our goal is, through a block randomization step and an optimization step, to minimize the difference between expected sample size *E*_*ij*_ and the actual sample size *n*_*ij*_.

The block randomization step is to create initial setup(s) of randomized sample assignment based on strata combining the blocking variables considered. The blocking variables include all variables of interests in the default algorithm, but only a specified subset of variables in the alternative algorithm.

In this step, we sample *i* sets of samples from each strata *S*_*j*_ with size ⌊*E*_*ij*_⌋, as well as *j* sets of wells from each *B*_*j*_ batches with size of ⌊*E*_*ij*_⌋. The two selections are linked together by the *ij* subgroup, randomized in each of them. The rest of samples *r*_*j*_ = *S*_*j*_ − ∑ _*i*_⌊*E*_*ij*_⌋ can be assigned to the available wells in each Block *w*_*i*_ = *B*_*i*_ − ∑ _*j*_⌊*E*_*ij*_⌋. The probability of a sample in *r*_*j*_ from strata *S*_*j*_ being assigned to a well from block *B*_*i*_ is proportional to the fractal part of the expected sample size *δ*_*ij*_. For a RCBD, each batch will have equal number of samples with same characteristic and there is no need for further optimization. However, for other instances where the collection of samples is unbalanced and/or incomplete, an optimization step is needed to create a more optimal setup of sample assignment.

The optimization step aims to identify an optimal setup of sample assignments from multiple candidates. To select optimal sample assignment, we need to measure the variation of sample characteristics between batches. In this package, we define the optimal design as a sample assignment setup that minimizes our objective function based on principle of least square method [13]. The objective function can be defined as

$$V=\sum_{\mathrm{ij}}{\left(n_{\mathrm{ij}}-E_{\mathrm{ij}}\right)}^{2}$$

where *E*_*ij*_ and *n*_*ij*_ were defined previously.

In the default algorithm implemented in OSAT, optimization is conducted through shuffling the initial setup obtained in the block randomization step. Specifically, after initial setup is created, we randomly select *k* samples from different batches and shuffle them between batches to create a new sample assignment. Value of the objective function is calculated for the new setup and compared to that of the original one. If the new value is smaller, the new assignment will replace the previous one. This procedure will continue until we reach a pre-set number of attempts (5000 by default).

In the alternative algorithm, multiple (typically thousands of or more) sample assignment setups are first generated by procedure described in the block randomization step above, based only on the list of specified blocking variable(s). The optimal one will be chosen by selecting the setup (from the pool generated in the block randomization step) which minimizes the value of the objective function based on all variables considered. This algorithm will guarantee the identification of a setup that is conformed to the blocking requirement for the list of specified blocking variables, while attempting to minimize the between-batches variations of the other variables considered.

## Implementation

We provide a brief overview of the OSAT usage as below. A more detailed description of package functionality can be found in the package vignette and manual.

### Data format

To begin, sample variables to be considered in the sample-to-batch assignment will be encapsulated in an object using function

sample<− setup.sample (x, optimal, …)where in data frame *x* each sample is represented by a row and category variables including our primary interest and other variables are listed as columns. The parameter *optimal* indicates the vector of variables to be considered.

### Batch layout

Next, the number of plates to be used in the genomic experiment, the layout design of these plates, and the level of batch effect to be considered are captured in a container object using constructor functionContainer <− setup.container(plate, n, batch, …)where parameter *plate* is an object representing the layout (number and type of chip used, rows and columns of wells, the ordering of them, and *etc.*) of the plate used in the experiment. Layouts of some commonly used plates and chips are predefined in our package (*e.g.*, the IlluminaBeadChip Plate). The user can define their own layout using the classes and methods provided in OSAT. Optional parameter *batch* has default value “plates”, indicate batch effect will be considered at the plate level. User can use *batch="chips"* to consider batch effect at chip level.

### Block randomization and optimization

Third, sample-to-batch assignment can be created through function

create.optimized.setup(fun="optimal.shuffle",sample, container, …)

The default algorithm is implemented in function *optimal.shuffle*, while the alternative algorithm is implemented in function *optimal.blcok*. Users can also define objective function following the instruction in the package vignette.

### Output

Last, bar plot of sample counts by batches for all variables considered is provided for visual inspection of the sample assignment. Chi-square tests are also to examine the dependence of sample variables on batches. The final sample-to-batch assignment can be output to CSV.

## Availability and requirements

Project name: OSATProject home page: http://bioconductor.org/packages/2.11/bioc/html/OSAT.htmlOperating system(s): Windows, Unix-like (Linux, Mac OSX)

Programming language: R >= 2.15

License: Artistic-2.0Any restrictions to use by non-academics: None

## Competing interests

The authors declare that they have no competing interests.

## Authors’ contributions

LY, CM and SL conceived and designed the study. LY developed the software. LY CM and SL drafted the manuscript. QH, DW, MQ, JMC, LES, CAB, CSJ and JW all contributed to the study design. All authors read and approved the final manuscript.

## Supplementary Material

Additional file 1 **Table S1.** Example data. **Table S2.** Data distribution. **Figure S1.** Number of samples per plate. Paired specimens are placed on the same chip. Sample assignment use optimal.block method. Click here for file
