# Supplementary material for: OSAT: a tool for sample-to-batch allocations in genomics experiments
Source: BMC Genomics. 2012 Dec 10;13:689. doi: 10.1186/1471-2164-13-689 (PMC3548766; doi:10.1186/1471-2164-13-689)
Supplement: Additional file 1 — Table S1. Example data. Table S2. Data distribution. Figure S1. Number of samples per plate. Paired specimens are placed on the same chip. Sample assignment use optimal.block method. [file 1471-2164-13-689-S1.pdf]

Supplementary material for: OSAT: a tool for sample-to-batch allocations in genomics experiments

Li Yan [1]*, Changxing Ma [2], Dan Wang [1], Qiang Hu [1] Maochun Qin,[1]
Jeffrey M. Conroy [3], Lara E. Sucheston [4], Christine B. Ambrosone [4],
Candace S. Johnson [5], Jianmin Wang,[1] and Song Liu [1]
[1]Department of Biostatistics,
[3]Cancer Genetics, [4]Prevention and Control, [5]Pharmacology and Therapeutics,
Roswell Park Cancer Institute, Buffalo, NY, 14263, USA
[2]Department of Biostatistics, University at Buffalo, Buffalo, NY, 14214, USA
`email:` li.yan@roswellpark.org

December 6, 2012

Contents

1 Example data **1**

2 Create an optimal sample assignment **2**

3 Alternative algorithms **6**
3.1 Potential problems with completely random assignment 7

4 Advanced usage **7**
4.1 Incomplete batches 7
4.2 Excluded well positions 8
4.3 Blocking and optimization on chip level 8
4.4 Predefined batch layout 8
4.5 Define your own batch layout 9
4.6 Define your own optimizing objective function 9
4.7 Paired samples 9

1 Example data

An example file is included for demonstration. It represent samples from a study where the primary interest is to investigate the expression differentiation in case versus control groups (variable `SampleType`). variables `Race` and `AgeGrp` are clinically important variables that may have impact on final outcome. We consider them as confounding variables. A total of 576 samples are included in the study, with one sample per row in the example file.

The data can be accessed by:

```
> library(OSAT)
> inPath <- system.file('extdata', package='OSAT')
> pheno <- read.table(file.path(inPath, 'samples.txt'),
+                     header=TRUE, sep="\t", colClasses="factor")
```

*to whom correspondence should be addressed

As shown in **Table 1**, the two groups in our primary interest `SampleType` are not balanced due to limitations in the sample collection process. To reduce possible batch effect across different plates, it is critical to make sure on each plate there are similar number of samples in each of the 2 stratas (Case/Control). Similarly, it is also preferred to have homogeneous makeup in each batch on confounding variables `Race/AgeGrp` which are not balanced as well. **Table 2** is the sample distribution when we included these three important variables considered.

Table 1: Example data

	ID	SampleType	Race	AgeGrp
1	1 : 1	Case :317	European:401	(0,30] : 75
2	10 : 1	Control:259	Hispanic:175	(30,40] :113
3	100 : 1			(40,50] :134
4	101 : 1			(50,60] :102
5	102 : 1			(60,100]:152
6	103 : 1			
7	(Other):570			

Table 2: Data distribution

	SampleType	Race	AgeGrp	Freq
1	Case	European	(0,30]	8
2	Control	European	(0,30]	58
3	Case	Hispanic	(0,30]	0
4	Control	Hispanic	(0,30]	9
5	Case	European	(30,40]	21
6	Control	European	(30,40]	54
7	Case	Hispanic	(30,40]	6
8	Control	Hispanic	(30,40]	32
9	Case	European	(40,50]	34
10	Control	European	(40,50]	52
11	Case	Hispanic	(40,50]	46
12	Control	Hispanic	(40,50]	2
13	Case	European	(50,60]	40
14	Control	European	(50,60]	44
15	Case	Hispanic	(50,60]	16
16	Control	Hispanic	(50,60]	2
17	Case	European	(60,100]	84
18	Control	European	(60,100]	6
19	Case	Hispanic	(60,100]	62
20	Control	Hispanic	(60,100]	0

2 Create an optimal sample assignment

We will use 6 Illumina 96-well HYP MultiBeadChip plates for this expression profiling experiment. Each plate will be processed at a time as a batch. This particular plate consists of 8 BeadChips, which in turn holds 12 wells in 6 rows and 2 columns. Each well holds an individual sample. We had predefined these and several other commonly used batch layouts (e.g., chips or plates) in the package.

First, sample pheno information and the variables considered (`i.e., SampleType, Race, AgeGrp`) can be captured by:

```
> gs <- setup.sample(pheno, optimal=c("SampleType", "Race", "AgeGrp"))
> gs
An object of class "gSample"
The raw data are
  ID SampleType     Race   AgeGrp
1  1       Case Hispanic (60,100]
2  2       Case Hispanic (60,100]
3  3       Case European (60,100]
4  4       Case European  (50,60]
5  5       Case European  (50,60]
6  6       Case European   (0,30]

 ...
     ID SampleType     Race  AgeGrp
571 571    Control European (40,50]
572 572    Control Hispanic (30,40]
573 573    Control European (30,40]
574 574    Control Hispanic (30,40]
575 575    Control European (40,50]
576 576    Control European  (0,30]

 Blocking strata in the data:

  SampleType Freq sFactor
1       Case  317       1
2    Control  259       2

 Optimization strata in the data

   SampleType     Race   AgeGrp Freq oFactor
1        Case European   (0,30]    8       1
2     Control European   (0,30]   58       2
3        Case Hispanic   (0,30]    0       3
4     Control Hispanic   (0,30]    9       4
5        Case European  (30,40]   21       5
6     Control European  (30,40]   54       6
7        Case Hispanic  (30,40]    6       7
8     Control Hispanic  (30,40]   32       8
9        Case European  (40,50]   34       9
10    Control European  (40,50]   52      10
11       Case Hispanic  (40,50]   46      11
12    Control Hispanic  (40,50]    2      12
13       Case European  (50,60]   40      13
14    Control European  (50,60]   44      14
15       Case Hispanic  (50,60]   16      15
16    Control Hispanic  (50,60]    2      16
17       Case European (60,100]   84      17
18    Control European (60,100]    6      18
19       Case Hispanic (60,100]   62      19
20    Control Hispanic (60,100]    0      20
```

A recommendation for practice is to put the variable of primary interest as the first of the list.

If pheno data is a phenoData slot in an `ExpressionSet` object `x` then the construction function should be called by:

```
setup.sample(pData(x), optimal, strata)
```

Second, 6 Illumina 96-well HYP MultiBeadChip plates were needed for the 576 samples. Each plate was treated as a batch. We will refer this assembly of plates (i.e., batches) as container.

```
> gc <- setup.container(IlluminaBeadChip96Plate, 6, batch='plates')
> gc
An object of class "gContainer"

It consists of 6 IlluminaBeadChip96Plate plates.
The block level is set at plates level.
  plates cFactor Freq
1      1       1   96
2      2       2   96
3      3       3   96
4      4       4   96
5      5       5   96
6      6       6   96
The container layout is
@data$container
  plates chipRows chipColumns chips rows columns wells chipID rowID wellID
1      1        1           1     1    1       1     1      1     1      1
2      1        1           1     1    2       1     2      1     1      2
3      1        1           1     1    3       1     3      1     1      3
4      1        1           1     1    4       1     4      1     1      4
5      1        1           1     1    5       1     5      1     1      5
6      1        1           1     1    6       1     6      1     1      6
  cFactor
1       1
2       1
3       1
4       1
5       1
6       1

 ...
    plates chipRows chipColumns chips rows columns wells chipID rowID wellID
571      6        2           4     8    1       2     7     48   286    571
572      6        2           4     8    2       2     8     48   286    572
573      6        2           4     8    3       2     9     48   286    573
574      6        2           4     8    4       2    10     48   286    574
575      6        2           4     8    5       2    11     48   286    575
576      6        2           4     8    6       2    12     48   286    576
    cFactor
571       6
572       6
573       6
574       6
575       6
576       6
```

`IlluminaBeadChip96Plate` is an predefined object in the package that represent the chips/wells layout of the Illumina 96-well HYP MultiBeadChip plates with 96 wells.

Third, samples will be assigned into container using our default allocation algorithm which consists of a block randomization step followed by an optimization step (this will take a few minutes):

```
> set.seed(123)    # to create reproducible result
> gSetup <- create.optimized.setup(sample=gs, container=gc, nSim=10000)
> gSetup
An object of class "gExperimentSetup"

Number of samples in each plate by blocking strata

     1  2
  1 53 43
  2 53 43
  3 53 43
  4 53 43
  5 52 44
  6 53 43

Number of samples in each plate by optimization strata

     1  2  3  4  5  6  7  8  9 10 11 12 13 14 15 16 17 18 19 20
  1  2 12  0  2  4  6  1  7  4  7  7  0  7  9  5  0 13  0 10  0
  2  1 11  0  1  4  8  1  4  7 10 10  0  6  9  2  0 17  0  5  0
  3  1  9  0  1  2  9  1  4  4 10  6  1  5  9  2  0 23  0  9  0
  4  1  8  0  3  4 10  0  5  5  7  6  1  7  8  3  1 10  0 17  0
  5  2  8  0  0  4 12  0  5  6  9  7  0 12  5  2  1  8  4 11  0
  6  1 10  0  2  3  9  3  7  8  9 10  0  3  4  2  0 13  2 10  0

The experiment setup is
@expSetup
  ID SampleType     Race   AgeGrp plates chipRows chipColumns chips rows
1  1       Case Hispanic (60,100]      3        2           2     6    1
2  2       Case Hispanic (60,100]      3        2           3     7    2
3  3       Case European (60,100]      6        2           3     7    2
  columns wells
1       2     7
2       2     8
3       2     8

 ...
     ID SampleType     Race  AgeGrp plates chipRows chipColumns chips rows
574 574    Control Hispanic (30,40]      1        1           1     1    1
575 575    Control European (40,50]      3        1           3     3    6
576 576    Control European  (0,30]      3        2           2     6    6
    columns wells
574       2     7
575       2    12
576       2    12
```

This is all one need to create an optimal sample-to-batch assignment using OSAT. All relevant output information is hold in the object `gSetup`. In above example, default algorithm `optimal.shuffle` is used so the parameter `fun=` is omitted.

The sample assignment can be output to CSV by:

```
> write.csv(get.experiment.setup(gSetup),
+           file="gSetup.csv", row.names=FALSE)
```

the output CSV file is sorted by the original row order from the sample list.

After the sample-to-batch assignment, a MSA-4 robotic loader can be used to load the samples onto the MultiBeadChip plate. If we are going to use a robotic loader, such as MSA 4 robotic loader, we can map our design to the loader and export the final lineup to a CSV file,

```
> out <- map.to.MSA(gSetup, MSA4.plate)
> write.csv(out, "MSAsetup.csv", row.names = FALSE)
```

the CSV file is sorted by the order used in the MSA robotic loader

A quick look at the sample distribution across the batches in our design created by OSAT:

```
> QC(gSetup)
```

The function `QC()` perform Pearson's Chi-squared test to examine the association between batches and each of the variables considered. The results indicate that all variables considered are all highly uncorrelated with batches (`p-value > 0.99`). Sample distribution by plates also is visualized, as shown in Figure 1 in the main article.

```
Test independence between "plates" and sample variables

 Pearson's Chi-squared test
         Var X-squared df   p.value
1 SampleType 0.2034518  5 0.9990763
2       Race 0.2380335  5 0.9986490
3     AgeGrp 0.8138166 20 1.0000000
```

3 Alternative algorithms

If blocking the primary variable (i.e., `SampleType`) is most important and the optimization of other variables considered are less important, a different algorithm can be used.

To demonstrate the difference of the two algorithms, we create a new optimal setup using the second method described above (this is slower than default method and will take a few minutes):

```
> gs2 <- setup.sample(pheno,  strata=c("SampleType"),
+           optimal=c("SampleType", "Race", "AgeGrp") )
> set.seed(123)    # to create reproducible result
> gSetup2 <- create.optimized.setup("optimal.block",
+           sample=gs2, container=gc, nSim=10000)
> QC(gSetup2)
```

```
Test independence between "plates" and sample variables

 Pearson's Chi-squared test
         Var  X-squared df   p.value
1 SampleType 0.03507789  5 0.9999879
2       Race 3.68541503  5 0.5955359
3     AgeGrp 5.08147313 20 0.9996856
```

Sample distribution by plates also is shown in Figure 2 in the main article.

Same functionality can be invoked by apply the optimizing objective function to any `gExperimentSetup` object directly:

```
gSetup1 <- optimal.shuffle(gSetup, nSim=10000)
gSetup2 <- optimal.block(gSetup, nSim=10000)
```

This syntax makes it easy to try different optimization methods and compare the results. Noticed that if `strata` parameter is not given in the constructor function `setup.sample()` to create the `gSample` object, the first variable in the `optimal` parameter will be used as the `strata` by default.

3.1 Potential problems with completely random assignment

Simply performing complete randomizations could lead to undesired sample-to-batch assignment, especially for unbalanced and/or incomplete sample sets. In fact, there is substantial chance that any one of the variables in the sample will be statistically dependent on batches if complete randomization is carried out.

For example, if we randomly assign our samples to the container, the innocent choice of a seed 397 in our R session will show:

```
set.seed(397)        # an unfortunate choice
c1 <- getLayout(gc)  # available wells
c1 <- c1[order(runif(nrow(c1))),] # shuffle randomly
randomSetup <- cbind(pheno, c1[1:nrow(pheno), ])
     # create a sample assignment
multi.barplot(randomSetup, grpVar='plates',
      varList=c("SampleType", "Race", "AgeGrp"),
      main="A bad random case")
multi.chisq.test(randomSetup, grpVar='plates',
      varList=c("SampleType", "Race", "AgeGrp"))
```

```
         Var X-squared df     p.value
 1 SampleType  13.25243  5 0.021124664
 2       Race  14.22455  5 0.014244218
 3     AgeGrp  39.75020 20 0.005371387
```

Pearson's Chi-squared test indicate all three variables are statistically dependent on batches with `p-values < 0.05`. Figure 3 in the main article shows the sample distribution in each plate based on this assignment.

4 Advanced usage

4.1 Incomplete batches

Since the number of wells available on a batch (e.g., plate/chip) is fixed, often experimenter cannot fill up all of them due to sample unavailability. In this scenario it is recommended that we distribute the unused wells randomly across all batches. This is done by default in our program without any additional instruction.

For example, assuming there are 10 samples not available for some reason in our sample list, the following create setup using the same container as before with the rest of 566 samples:

```
> # for demonstration, assume 10 bad samples
> badSample <- sample(576, 10, replace=FALSE)
> # create sample object using available samples
> gs3 <- setup.sample(pheno[-badSample, ],
+       optimal=c("SampleType", "Race", "AgeGrp"),
+       strata=c("SampleType") )
> # use the same container setup as before
> gSetup3 <- create.optimized.setup(sample=gs3,
+         container=gc, nSim=10000)
```

To fill batches sequentially and have the empty wells left at the end of last plate is discouraged. This practice will create a batch that is immediately different from the others, making statistical analysis more complex and less powerful. However, if there are strong reasons to do so, we can exclude the wells at the end of last plate for sample assignment, using method described in the following section.

4.2 Excluded well positions

If for any reason we need to reserve certain wells for other usage, we can exclude them from the sample assignment process. For this one can create a data frame to mark these excluded wells. Any wells in the container can be identified by its location identified by three variable "plates", "chips", "wells". Therefore the data frame for the excluded wells should have these three columns.

For example, if we will use the first well of the first chip on each plate to hold QC samples, these wells will not be available for sample placement. We have 6 plates in our example so the following will reserve the 6 wells from sample assignment:

```
> excludedWells <- data.frame(plates=1:6, chips=rep(1,6),
+                   wells=rep(1,6))
```

Our program can let you exclude multiple wells at the same position of plate/chip. For example, the following data frame will exclude the first well on each chips regardless how many plates we have:

```
> ex2 <- data.frame(wells=1)
```

In our example we have 48 chips so 48 wells will be excluded using this data frame for exclusion. We can pass our exclusion data frame to the container setup function using this command:

```
> gc3 <- setup.container(IlluminaBeadChip96Plate, 6,
+              batch='plates', exclude=excludedWells)
```

4.3 Blocking and optimization on chip level

In certain circumstances, batch effects between chips maybe considered. This task can be accomplished by simply indicate the level of block in the container construction, for example

```
> cnt <- setup.container(IlluminaBeadChip96Plate, 2, batch='chips')
```

will create a container with 16 chips. Blocking and optimization on chip level will be used in following sample assignment.

4.4 Predefined batch layout

Function `predefined()` shows a list of predefined object that represent some of commonly used plates and chips. Simply typing the name in R shows the layout and other information of each predefined chips and plates.

IlluminaBeadChip is a chip with 2 columns and 6 rows, a total of 12 wells:

```
> IlluminaBeadChip
An object of class "BeadChip"
Illumina Bead Chip has 6 rows and 2 columns.
The layout is
@layout
rows columns wells
1 1 1 1
2 2 1 2
3 3 1 3
4 4 1 4
5 5 1 5
6 6 1 6
7 1 2 7
8 2 2 8
9 3 2 9
10 4 2 10
```

```
11 5 2 11
12 6 2 12
```

IlluminaGenotypingChip is a chip with 1 columns and 12 rows.

IlluminaBeadChip24Plate, IlluminaBeadChip48Plate, IlluminaBeadChip96Plate are plates that hold 2, 4, 8 `IlluminaBeadChip` chips and have 24, 48, 96 wells, respectively.

4.5 Define your own batch layout

As long as the physical layout is know for a plate or chip, it is straight forward to define your own object in this package. For example, if you have a chip that has 12 wells arranged by a 2 columns and 6 rows pattern, and the index of the wells are filled columns by columns, then we can create (and have a look at) a new chip object simply:

```
> myChip <- new("BeadChip", nRows=6, nColumns=2, byrow=FALSE,
+       comment="Illumina Bead Chip have 6 rows and 2 columns.")
```

The `byrow` parameter is same as that in function `matrix()`. The chip happens to be identical to the `IlluminaBeadChip` defined in the package.

Similarly, we can define plate simply based on the chips it contains. Here we create a new plate that consistent 8 `myChip` we just defined:

```
> myPlate <- new("BeadPlate", chip=IlluminaBeadChip,
+       nRows=2L, nColumns=4L,
+       comment="Illumina BeadChip Plate with 8 chips and 96 wells")
```

it happens to be identical to the `IlluminaBeadChip96Plate` plate defined in the package.

4.6 Define your own optimizing objective function

Our package allows user to define optimization objective function themselves. It is straightforward for user to define their own optimization objective function, following a few parameter requirements for the package to work. In particular, the objective function parameters should include a `gExperimentSetup` object, and number of loops used in the form of

```
myFun <- function(x, nSim, ...)
```

It can include additional parameters for its own in . . . parameter list.

Also, the function should return a list that including following elements:

```
return(list(setup=, link=, average=, dev=))
```

where elements `setup` is a `gExperimentSetup` object, `link` is a data frame present a link between the sample and container, `average` is the expected number of samples from each blocking strata in each of the optimization blocks, `dev` a vector of values created by the actual objective function. Using these guideline, user can use the helper functions in the package such as `QC` to inspect the result.

4.7 Paired samples

OSAT can be used to handle experiment design with paired examples.

Assuming each individual listed in our `samples.txt` file had 2 specimens. One is collected prior certain treatment and the other after. We would like to keep the two specimens on the same chip, to reduce the batch effect on the treatment effect. Other requirements are similar to those in the section 1.

To accomplish this new task, one chip can only hold specimens from 6 individuals. We would first assign specimen pairs onto chips, then shuffle them within each chip randomly to further eliminate potential location bias within chips. To do this, we will create a mock chip that only has 6 rows and 1 column instead of 2

columns. Each row on this mock chip will be assign to one individual. Plate and container are created based on this new chip. After assignment, we will expand the chips to 2 columns.

First of all the pairs are assigned into rows of the mock chip (noticed now we need 12 plates):

```
# create mock chip. each row represent one individual
newChip <- new("BeadChip",  nRows=6, nColumns=1, byrow=FALSE,
   comment="mock chip")
# a mock plate based on above chip, same physical layout
newPlate <- new("BeadPlate", chip=newChip,
   nRows=2L, nColumns=4L,
   comment="mock plate")
# create containers based on above mock chip/plate
gcNew <- setup.container(newPlate, 12, batch="plates")

# assign pairs into locations on the mock chip
# this will take some time
set.seed(123)
gPaired <- create.optimized.setup("optimal.block", sample=gs,
           container=gcNew, nSim=10000)
```

The above steps create sample setup that place paired sample to rows of each real BeadChip, the following steps expand the chips to real Illumina BeadChip. First assign specimens into first column on each real chip:

```
set.seed(456)
out1 <- get.experiment.setup(gPaired)
out1$Replica <- FALSE
idx <- sample(nrow(out1), ceiling(nrow(out1)/2), replace=FALSE)
     # randomly decided if the first specimen is placed in column 1
out1[idx, "Replica"] <- TRUE
summary(out1$Replica)
```

above we randomly selected half of before-treatment specimens and half of after-treatment specimens into first the column. The rest specimens will be placed into the second column:

```
out2 <- out1
out2$columns <- 2          # specimen placed in the second column
out2$wells <- out2$wells+6 # correct well number
out2$Replica <- !out1$Replica          # indicate second specimen

out3 <- rbind(out1, out2)  # all specimens

# sort to order on plates/chips/rows
idx1 <- with(out3, order(plates, chips,  rows, columns, wells))
out3 <- out3[idx1,]  # sort to order on plates/chips/wells
head(out3,12)
```

This procedure will place paired specimens on the same row of the same chip. If do not want to keep pairs of specimens on the same row, we can shuffle one more time:

```
## shuffle within chip
set.seed(789)
idx2 <- with(out3, order(plates, chips, runif(nrow(out3))))
out4 <- cbind(out3[, 1:8], out3[idx2, 9:11], Replica=out3[, 12])

# sort to order on plates/chips/wells
idx3 <- with(out4, order(plates,  chips, wells))
out4 <- out4[idx3,]
head(out4,12)
```

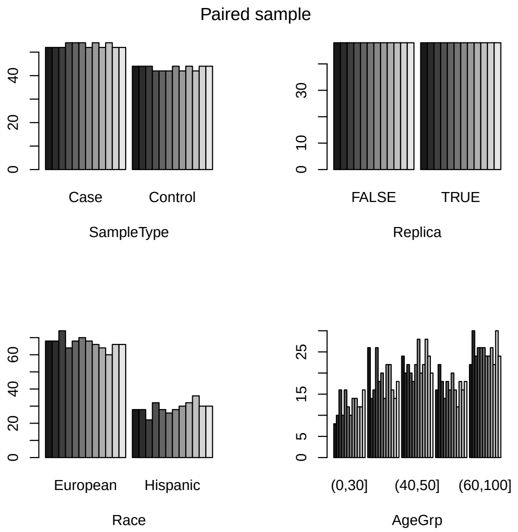

Figure 1: Number of samples per plate. Paired specimens are placed on the same chip. Sample assignment use `optimal.block` method.

Check the assignment:

```
# SampleType and replica distribution by plate
ftable(xtabs( ~plates +SampleType+ Replica, out3))

                  Replica FALSE TRUE
plates SampleType
1      Case                  26   26
       Control               22   22
2      Case                  27   27
       Control               21   21
3      Case                  27   27
       Control               21   21
...
```

The final assignment quality can be checked by Pearson Chisq test and visualized by bar plot:

```
multi.barplot(out3, grpVar='plates', varList=c("SampleType", "Replica",
              "Race", "AgeGrp"), main="paired sample")
multi.chisq.test(out3, grpVar='plates', varList=c("SampleType", "Replica",
              "Race", "AgeGrp"))

         Var  X-squared df   p.value
1 SampleType  0.4910905 11 0.9999988
2    Replica  0.0000000 11 1.0000000
3       Race  6.4843605 11 0.8391738
4     AgeGrp 30.0921186 44 0.9455054
```

The Pearson's test and Figure S1 show perfect `Replica` balance between plates due to the fact all paired specimens are placed on the same chip.
